# Temporary Life Changes and the Timing of Divorce

**DOI:** 10.1007/s13524-016-0498-2

**Published:** 2016-09-13

**Authors:** Peter Fallesen, Richard Breen

**Affiliations:** 1 Swedish Institute for Social Research, Stockholm University, SE-106 91 Stockholm, Sweden; 2 Rockwool Foundation Research Unit, Copenhagen, Denmark; 3 Nuffield College, Oxford University, Oxford, United Kingdom

**Keywords:** Children, Divorce, Information processes, Relationship disruption, Uncertainty

## Abstract

Marriage is a risky undertaking that people enter with incomplete information about their partner and their future life circumstances. A large literature has shown how new information gained from unforeseen but long-lasting or permanent changes in life circumstances may trigger a divorce. We extend this literature by considering how information gained from a temporary change in life circumstances—in our case, a couple having a child with infantile colic—may affect divorce behavior. Although persistent life changes are known to induce divorce, we argue that a temporary stressful situation allows couples more quickly to discern the quality of their relationship, in some cases leading them to divorce sooner than they otherwise would have. We formalize this argument in a model of Bayesian updating and test it using data from Denmark. We find that the incidence of infantile colic shortens the time to divorce or disruption among couples who would have split up anyway.

I see it is a good thing for husband and wife to have the occasional disagreement, since they thereby come to learn things about one another.

Johann Wolfgang von Goethe (1809/1978)

## Introduction

Marriage is an inherently risky undertaking. People cannot search indefinitely for the perfect partner because searching is costly and time consuming (Mortensen 1988). Thus, when people do marry, they are understandably uncertain about the qualities and characteristics of their partner and their marriage. Partners continue to learn about their spouse and their relationship after marriage—a process that may end in disappointment and divorce.

The literature suggests two main, broad causes of divorce (see Lyngstad and Jalovaara 2010 for a review). First, because of the costs of searching, few (if any) marriages will be the best possible match that both partners could have made. Mate search does not always end at marriage. With the advent of new information, a better partner might be discovered (Becker et al. 1977). For example, an individual could learn about potential partners through the inflow of new colleagues into the workplace (McKinnish 2004, 2007). The second cause of divorce is unforeseen changes in the characteristics of the partner or the relationship, leading to a change in the perceived (and actual) value of the marriage. Weiss and Willis examined the role of unanticipated changes in the earnings capacities of husbands and wives in the United States to investigate how the arrival of new information (i.e., not available at the time of the marriage) may trigger divorce (Weiss and Willis 1997). Similarly, other research has pointed to the birth of a child with a handicap or behavioral disorder as a source of increased divorce risk (e.g., Kvist et al. 2013; Schermerhorn et al. 2012). In both cases, a change in circumstances brings about a long-term, or even permanent, change in the nature of the marital relationship.

In this article, we consider a third possible use of information to inform the divorce decision. At any point in time, a marriage has a particular quality about which the partners hold beliefs that may be more or less accurate. Previous research has focused on how events may change this quality. Here, we focus instead on the role of events that change the partners’ beliefs but do not change the underlying quality itself. In particular, we consider how events lead the partners to update their beliefs about the quality of the marriage and thus how this affects the likelihood of divorce. The specific event that we consider—having an infant suffering from colic—is both random and temporary (Savino et al. 2013), and it has no consequences for the economic gains to marriage or long-term expected utility of the relationship (Stifter and Bono 1998). Instead, parents’ responses to the stress caused by having an infant with colic provide information about the quality of the marriage (Brüderl and Kalter 2001). We show that having a child suffering from colic accelerates but does not increase the incidence of divorce, implying that the colic event does not change the quality of the marriage but does affect the rate at which husbands and wives learn about the quality of their marriage.^1^

## Learning and Divorce

In their seminal study of marital instability, Becker et al. (1977) argued that people divorce when they expect higher economic gains by reentering the marriage market rather than staying in their present marriage. An important element of the Becker et al. model, and of many subsequent models of marriage and divorce, is that people marry without complete information about the nature of their relationship. Thus, marriage is, among other things, a process of learning about one’s partner and one’s marriage. In the literature, learning takes two main forms. On the one hand, even if partners had complete knowledge of their relationship at marriage, unforeseen events may occur that change the nature of that relationship. Weiss and Willis (1997), for example, considered unanticipated shocks to the spouses’ earnings capacity. On the other hand, in reality, partners do not have complete knowledge of their relationship at marriage, and so they learn about aspects of it that, in an ideal world, they would have known before marriage (Brüderl and Kalter 2001). One way (albeit oversimplifying) of capturing the distinction is to say that in the first case, partners are learning about time-varying characteristics of their relationship, but in the latter, they are learning about invariant qualities of their relationship. Another way of capturing the difference is to say that in the first case, learning concerns observables, but in the second case, learning concerns unobservable attributes of the relationship (see Weiss and Willis 1997:S320, and S300, equation 1).^2^

Within marriage, partners constantly update their beliefs about their relationship, but this learning or updating is usually slow because the information supplied by the partner’s actions is mostly of relatively low quality. The receipt of higher-quality information will speed up the learning process. Yet, because learning in this case involves the unobserved relationship quality, it will not affect the belief to which the learning process will converge. That is, partners in a mutually compatible relationship will learn that they are compatible more quickly than they would otherwise, and similarly for partners in a mutually incompatible relationship. We show that infantile colic^3^ is a shock that provides much high-quality information to partners in a relatively short time and thus accelerates their learning about the real nature of their relationship, thus leading couples who would in any case divorce, to divorce sooner.

Between 10 % and 30 % of all infants suffer from colic between the ages of 3 and 6 months (Savino 2007). The main symptom of colic is excessive and persistent crying. The “rule of three” establishes the criteria for diagnosing colic: crying in an otherwise healthy infant (1) for at least three hours per day, (2) at least three days per week, (3) for at least three weeks (Brazelton 1962; Wessel et al. 1954). Infantile colic places the parents’ relationship under increased strain, and couples whose newborn child suffers from colic have higher divorce and disruption rates and report higher dissatisfaction with family life in the years immediately following the colic (Rautava et al. 1995; Tanner 2002; Vik et al. 2009). However, colic does not appear to have long-term health consequences for children (Canivet et al. 2000), and it does not seem to cause persistent changes in family life (Clifford et al. 2002; Stifter and Bono 1998).

Researchers know little about the causes of colic, and it appears to occur randomly,^4^ with general agreement that it is not hereditary (Savino et al. 2013). The uncertainty about causes results in a number of suggested remedies, but no therapeutic approach has been shown to be more effective than a placebo (Garrison and Christakis 2000; Roberts et al. 2004; Savino et al. 2013). Parents may know from its onset that colic is a passing affliction; nevertheless, the temporary disruption forces them to cooperate more than normal in order to maintain a functioning everyday life. An episode of colic may call for greater bargaining about the division of labor both inside and outside the home, which might lead to a higher level of conflict, making it clearer on what issues the couple disagrees. We argue that in this situation, couples will gain better insight into each other’s values and priorities and learn more quickly about the nature of their relationship. We formalize these ideas in a model of learning and divorce.

### A Model of Learning and Divorce

A couple is assumed to be one of two types: compatible (*C*) or incompatible (*I*). Each couple’s^5^ type is fixed and does not change, but couples do not know their type at marriage. Instead, they have a belief, *p*, which is the weight they assign to *C* being the true state of their relationship; thus, Pr(*C*) = *p*. At marriage, each couple has their own unique initial belief, *p*_0_, which they update during their marriage using signals that they receive from each other’s actions. These signals can be positive/good (*G*) or negative (*N*) and are informative about the true state of the relationship because a positive signal is more likely if the true state is *C*, and a negative signal is more likely if the true state is *I*. We can define

$$ r= \Pr \left(\mathrm{signal}\ \mathrm{is}\ G\left|\mathrm{couple}\ \mathrm{is}\ C\right.\right) $$

and

$$ s= \Pr \left(\mathrm{signal}\ \mathrm{is}\ N\left|\mathrm{couple}\ \mathrm{is}\ I\right.\right), $$

and we assume that *r >* 1/2 and *s >* 1/2. The parameters *r* and *s* tell us the information content of the signals. The higher the values of *r* and *s*, the more informative the signal.

A couple receives one signal per period, *t*, and their beliefs evolve using Bayesian updating as follows:

$$ {p}_{t+1}=\frac{p_tr}{p_tr+\left(1 - {p}_t\right)\left(1 - s\right)}\ \mathrm{if}\ \mathrm{the}\ \mathrm{signal}\ \mathrm{is}\ G $$

and

$$ {p}_{t + 1}=\frac{p_t\left(1 - r\right)}{p_t\left(1 - r\right)+\left(1 - {p}_t\right)s}\ \mathrm{if}\ \mathrm{the}\ \mathrm{signal}\ \mathrm{is}\ N. $$

In each period, the couple decides whether to stay together or divorce. To make this decision, they compare the payoff from divorce, *U*(*D*), with the payoff from remaining married, *U*(*M*). The benefits of remaining married depend on whether the couple is compatible or incompatible; thus:

1 $$ U{(M)}_t={p}_tU(C)+\left(1 - {p}_t\right)U(I). $$

The payoff from remaining together in a given period depends on the payoffs from a compatible and incompatible marriage, respectively, weighted by the current belief. We assume that *U*(*D*), *U*(*C*), and *U*(*I*) are fixed over time for a given couple,^6^ but they can differ between couples. However, for all couples, *U*(*C*) > *U*(*D*) ≥ *U*(*I*).

A couple divorces in period *t* if

$$ U(D)>U{(M)}_t. $$

that is,

2 $$ U(D)>{p}_tU(C)+\left(1 - {p}_t\right)U(I). $$

From Eq. (2), it follows that each couple has a threshold belief, ${p}^{*}$, such that when their belief (*p*) reaches ${p}^{*}$, they will, all things equal, divorce. The threshold is given by

$$ {p}^{*}=\frac{U(D) - U(I)}{U(C) - U(I)}. $$

Notice that we must have *p*_0_ > ${p}^{*}$ (an initial belief above the threshold) in order for a couple to marry in the first place. Whether, and how soon, a couple’s belief reaches their threshold and leads to divorce depends on (1) their particular initial belief; (2) the location of their threshold (which depends on their particular values of *U*(*D*), *U*(*C*), and *U*(*I*)); and (3) how informative are the signals they receive (given by *r* and *s*).^7^

The dynamics of the process are simple. If the couple’s true state is *I*, their threshold will eventually be reached: truly incompatible couples will eventually divorce (assuming that they have the possibility and the opportunity). A couple whose true state is *C* may, nevertheless, divorce if their initial belief, *p*_0_, is close to their threshold. This situation may arise because the threshold itself is relatively high (e.g., they might have very similar payoffs from divorce and from remaining with a compatible partner) or because their initial belief was very wide of the mark. In such cases, *p*_*t*_ could fall below p* because of a string of unlucky *N* signals. Although a compatible couple would be more likely to receive the *G* than the *N* signal, they might, by chance, receive a string of *N* signals sufficient to push their value of *p* below their threshold, leading them to (mistakenly) divorce.^8^

The probability that *p*_*t*_ for a compatible couple reaches ${p}^{*}$, given that *p*_0_ > ${p}^{*}$, is equal to $\frac{p^{*}\left(1 - {p}_0\right)}{p_0\left(1 - {p}^{*}\right)}=\frac{p^{*} - {p}^{*}{p}_0}{p_0 - {p}^{*}{p}_0}$ (see Breen and García-Peñalosa 2002:910, 918–919). From this, it is evident that the probability of a compatible couple divorcing declines as *p*_0_ becomes more distant from the threshold belief.

For an incompatible couple, because both *p*_0_ and ${p}^{*}$ are fixed—and given that such couples are more likely to receive negative than positive signals (*s* > 1/2)—the timing of their divorce depends solely on the degree of information conveyed by those signals. High-information signals will hasten divorce, whereas low-information signals will delay it.

## Informative Signals

Some signals are more informative than others. Discussing the division of household labor when expecting a child or after recently having one might provide a large amount of new information (see Hochschild 1989), whereas stating one’s dinner preferences would provide very little. Conflict could also yield clearer signals of values and preferences. Transitory shocks to the life situation, such as short periods of stress like that caused by infantile colic, can lead to the production of highly informative signals. Nevertheless, the extent to which the information that is revealed in this way is new will depend on how much information couples already have. Couples who, under normal circumstances, have negotiated more will already have a good estimate of whether they are compatible, and so we should expect new information to have a smaller impact on their beliefs. Similarly, new information will have a smaller impact on couples who have been together longer, perhaps partly because incompatible couples will already have divorced, leading to a selection of more compatible couples into longer relationships, and partly because couples who have been together longer have had more time to receive signals and have more robust beliefs about each other (Brüderl and Kalter 2001). In both cases, the relative value of informative signals, such as those associated with a spate of colic, will decrease as the amount of information already acquired by the time of the signal increases.

## Empirical Application

To test the learning model, we use a Danish panel data set of 4,920 couples who had a child in 1995. We follow their relationship status until 2006. We consider colic as a randomized shock whose incidence generates highly informative signals about the partners’ relationship. In line with our previous discussion, we show that having an infant with colic shortens the time to divorce but has a weaker effect on couples who had been together longer prior to the birth of the colicky child and also on couples who already had children.

### Data

We use the Danish Longitudinal Survey of Children (DALSC) to examine how infantile colic affects divorce and relationship disruption. DALSC randomly sampled more than 6,000 Danish children born in 1995. Interviewers conducted a first postnatal interview with the mother when the child was 6 months old. We focus on children whose parents responded to the survey and reported living with their partner when the child was born, giving a sample of 4,920 couples. We can link children to their parents through the child’s Social Security number, and we use administrative data to track the parents’ relationship status annually from January 1, 1996, to January 1, 2006. We define two people as being in a relationship if they are living at the same address (Danish citizens are required by law to give notice within five days of changing their address) and both are the registered birth parents of the child. The measure is very reliable: indeed, it is used by Statistics Denmark when reporting Danish children’s living arrangements (Petersen and Nilesen 2008).

Our definition of relationship status means that we treat married and cohabiting couples the same. Whereas family demographers, especially in the United States, have tended to view cohabitation as a separate and less-stable form of relationship than marriage (e.g., Axinn and Thornton 1992; Smock 2000; Tach and Edin 2013), ample evidence exists from Northern European countries that cohabiting couples are, or are becoming, similar to married couples (see Heuveline and Timberlake 2004; Lundberg 2005; Svarer 2004; Verbakel and Kalmijn 2014). In additional analysis (omitted for the sake of brevity but available on request), we find no evidence that colic affects the timing of relationship dissolution differently for cohabiting compared with married parents, although cohabiting parents do have a higher overall dissolution rate. Of necessity, our empirical analysis deals only with couples with children, and this method likely contributes to the lack of difference that we find between married and cohabiting couples. When choosing to have a child, most couples will (we assume) hold beliefs about their compatibility that are far above their threshold: in other words, we assume that couples will usually have a child only if they believe they are compatible.^9^

Table 1 presents descriptive statistics for all Danish women who had a child in 1995, for the full DALSC sample, and for the specific sample used in the analysis. We focus on women because they were the primary respondents in the DALSC survey. Compared with the average Danish woman who gave birth in 1995, the women in the full DALSC sample and the estimation sample were more likely to have another child prior to the one born in 1995, to be in a relationship at the beginning of 1995, and to be older when giving birth. Both DALSC groups also had slightly more education than the average woman giving birth in 1995, and their children weighed more at birth. Previous research from other Nordic countries has found a negative relationship between educational attainment and divorce risk (Härkönen and Dronkers 2006; Lyngstad 2004). Although we not only consider divorce but also relationship dissolution, the aforementioned studies suggest that our estimates may understate the effect of infantile colic on divorce and dissolution in the general population.

**Table 1:** Descriptive statistics for sample compared with average Danish parents who had a child in 1995

	Danish Population	Full DALSC	Estimation Sample	*p* Value Population vs. Full	*p* Value Population vs. Sample	*p* Value Full vs. Sample
Older Siblings	.566	.618	.580	.001	.001	.001
	(.496)	(.486)	(.494)			
In Relationship as of January 1, 1995	.854	.926	.968	.001	.001	.001
	(.353)	(.261)	(.176)			
Birth Weight (kg)	3.484^a^	3.510^c^	3.520^f^	.001	.001	ns
	(0.598)	(0.605)	(0.603)			
Mother’s Age at Birth	28.250	30.173^d^	29.315	.001	.001	.001
	(4.688)	(4.674)	(4.552)			
Mother’s Years of Education	12.312^b^	12.491^e^	12.634^g^	.001	.001	.001
	(2.272)	(2.200)	(2.157)			
Infantile Colic		.133	.146			ns
		(.340)	(.353)			
Smoking When Child Is 6 Months		.277	.294			ns
		(.448)	(.456)			
Relationship Length in Years			7.345			
			(4.163)			
*N*	67,657	5,666	4,920			

^a^ *N =* 67,080.

^b^ *N* = 66,437.

^c^ *N =* 5,124.

^d^ *N* = 5,588.

^e^ *N =* 5,552.

^f^ *N =* 4,919.

^g^ *N =* 4,854.

### Infantile Colic as an Informative Signal

To test how an informative signal affects the timing of divorce and dissolution, we use the random nature of infantile colic. The only identified predictor of colic is prenatal exposure to nicotine (Milidou et al. 2012); otherwise, neither parents nor medical professionals can predict whether a child will develop colic. Couples whose newborn child suffers from colic have higher divorce and disruption rates and report more relationship distress in the time following the colic (e.g., Tanner 2002; Vik et al. 2009). However, colic is a passing affliction that does not have long-term behavioral effects on children.^10^ Although previous research has found colic to be associated with short-term maternal depression (Vik et al. 2009) and poorer infant-mother attachment during the first months of infancy (Stifter and Bono 1998), these effects are transitory; research has found infantile colic to have no lasting effects on maternal mental health (Clifford et al. 2002; Stifter and Bono 1998).

Interviewers asked respondents in DALSC whether the child had suffered from infantile colic. Although our information about the incidence of colic comes from the respondent, we have good reasons to believe that it is accurate. All Danish mothers receive home visits by nurses within days of giving birth as well as future visits in the child’s first months (the exact number of visits varies between municipalities): 99.4 % of all respondents in our sample had received at least one visit. Furthermore, the “rule of three” is an exact and widely known definition of colic. The regular home visiting scheme, the clear definition, and the fact that the interview occurred when the child was 6 months old lead us to expect our colic measure to be highly reliable. In addition, the reported incidence of colic in our data (14.6 %) is well within the expected range (Savino 2007).

**Fig. 1:**
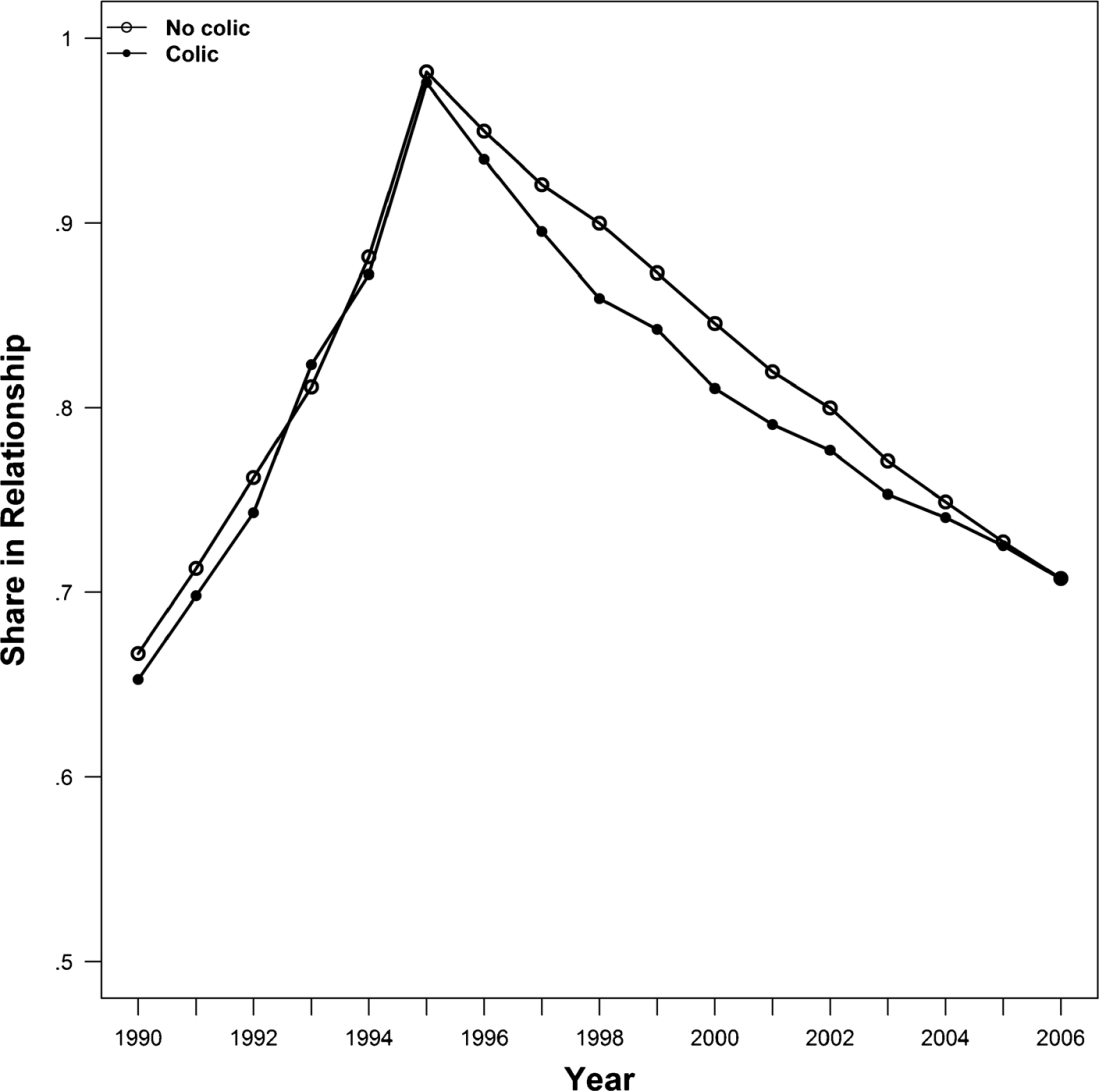
Union rates for sample couples with versus those without an infant with colic, 1990–2006. *Source:* DALSC and own calculations on data from Statistics Denmark

Figure 1 shows the union rates for couples whose child had infantile colic and for couples whose child did not. Before the birth of the child, there was no discernible difference in relationship rates for the two groups. After 1995, couples whose child suffered from infantile colic dissolved their relationship at a faster rate than couples whose child did not. Yet, by 2005, 10 years after the birth of the child, the shares of the two groups still in the same relationship were identical. Thus, couples with colicky infants dissolved their relationships faster than couples with noncolicky infants, but colic was not associated with a higher divorce rate over the entire 11-year period that we consider.

If colic is, as argued earlier, a random event (conditional on the use of nicotine products during pregnancy, as we noted earlier), then plausible covariates should not have explanatory power when we regress the incidence of colic on them. Therefore, we run the following regression model (suppressing subscripts):

3 $$ \begin{array}{c}\hfill \Pr \left( Colic=1\right)={\upbeta}_0+{\upbeta}_1 Smoking+{\upbeta}_2 \log \left( Relationship\kern0.5em  length\right)+{\upbeta}_3 Siblings+{\upbeta}_4 Birth\kern0.5em  weight\hfill \\ {}\hfill + {\upbeta}_5 Mother's\kern0.5em  age\kern0.5em  at\kern0.5em  childbirth+{\upbeta}_6 Mother's\kern0.5em  years\kern0.5em  of\kern0.5em  education+\upvarepsilon .\hfill \end{array} $$

Table 2 shows results of running Eq. (3) using ordinary least squares (OLS) regression. We regress the colic indicator on whether the mother smoked six months after giving birth (our best proxy for smoking during pregnancy), log of relationship length prior to birth, a sibling dummy variable indicating that the couple already had children, child’s birth weight in kilos, mother’s age at birth, and mother’s years of education. The model was fitted using data on all couples who were in a relationship the year the child was born (1995). As expected, smoking is a significant predictor, increasing the probability of having a colicky infant by 2.6 percentage points. Whether the child is the firstborn or has siblings also matters. Parents who already had a child had a 3.5 percentage point lower probability of reporting infantile colic. A possible explanation is that if parents are uncertain about whether the child has colic, those who already have children might be more likely to respond negatively because they have a higher threshold for when persistent crying should be considered to be colic. We control for smoking and whether the child was the firstborn in our subsequent models. We also run separate analyses for first-time parents because infantile colic might be a more informative signal for them than for couples who already had children.

**Table 2:** Estimates of predictors for colic: Results from linear probability model

	Coefficient
Smoking	0.026*
	(0.012)
Older Siblings	–0.035**
	(0.012)
log(Relationship Length)	0.017
	(0.009)
Birth Weight (kg)	–0.016
	(0.009)
Mother’s Age at Birth	–0.002
	(0.001)
Mother’s Years of Education	–0.002
	(0.003)
Constant	3.368
	(2.627)
*R* ^2^	.004
*F* Value	3.18**
*N*	4,737

*Notes:* Standard deviations are shown in parentheses.

*Source:* DALSC and own calculations on data from Statistics Denmark.

*p < .05; **p < .01

### Predicting Divorce and Dissolution

To examine how having an infant with colic affects the incidence and timing of divorce among couples with children, we estimate annual linear probability models for the 11 years following the child’s birth (1996–2006), regressing a dummy variable for whether the parents were together on a set of predictors. We control for smoking as a confounder affecting both colic and the risk of divorce, and we allow the impact of colic to differ across relationship length. We specify the model as follows (suppressing subscripts):

4 $$ \begin{array}{l} \Pr \left( Relationship=1\right)={\upbeta}_0+{\upbeta}_1 Colic+{\upbeta}_2 log\left( Relationship\  length\right)+{\upbeta}_4 Smoking\kern6.5em \\ {} + {\upbeta}_3 Colic\times log\left( Relationship\  length\right)+{\upbeta}_5 Smoking\times Colic+{\upbeta}_6 Siblings+\upvarepsilon .\end{array} $$

*Colic* is an indicator of whether the focal child (born in 1995) had colic. *Relationship length* measures the number of years a couple cohabited prior to the birth of the focal child. *Smoking* is an indicator of whether the mother smoked six months after giving birth. *Siblings* indicates whether the focal child has older siblings. The interaction between infantile colic and relationship length captures the idea that signals have less effect on couples who have been together longer. We log-transform relationship length to allow the value of any single signal to decrease as couples accumulate information over time.^11^ We interact colic and smoking to capture possible confounding because smoking increases the risk of colic but is also likely an indicator of other unobserved characteristics, such as unhealthy lifestyles and low time discounting, that might affect the risk of relationship dissolution. Table 2 indicates that having previous children (Siblings = 1) also affects colic risk. Instead of interacting colic with the sibling indicator, we present results from subsamples defined by whether the focal child is the parents’ firstborn. Because having a child itself likely also produces highly informative signals, parents who already have children learn less from having a colicky child. We use the linear probability model to avoid problems of noncomparability of coefficient estimates that arise when we use nonlinear probability models, such as the logit or probit (Breen et al. 2014; Mood 2010).

## Findings

Table 3 reports the annual predictors of staying in a union for the period 1996–2006. All couples in the sample were living together at the start of 1995. We measure all the explanatory variables at the time of childbirth, so they remain constant across years. Thus, the variation in regression coefficients is due to changes in the couples’ relationship status.^12^ Infantile colic significantly increased the risk of not being in a relationship in the years 1997–2004, while the estimates for colic are smaller and insignificant for 1996, 2005, and 2006. The likelihood of still being together was lower in all years for couples in which the woman smoked during pregnancy. For the period 2001–2004, we also find significant and positive parameter estimates for the colic-smoking interaction, indicating some confounding by maternal smoking.

**Table 3:** Estimates of probability of remaining in union for couples with a colicky infant and those without, 1996–2006

	1996	1997	1998	1999	2000	2001	2002	2003	2004	2005	2006
Colic	–0.072 (0.040)	–0.125** (0.047)	–0.152** (0.050)	–0.151** (0.053)	–0.148** (0.056)	–0.171** (0.057)	–0.170** (0.057)	–0.122* (0.059)	–0.125* (.059)	–0.086 (0.060)	–0.076 (0.060)
log(Relationship Length)	0.051*** (0.007)	0.079*** (0.008)	0.083*** (0.008)	0.101*** (0.009)	0.111*** (0.010)	0.125*** (0.0104)	0.130*** (0.011)	0.138*** (0.011)	0.134*** (0.011)	0.138*** (.012)	0.141*** (0.011)
Colic × log(Relationship Length)	0.032 (0.019)	0.051* (.022)	0.058* (0.023)	0.056* (0.025)	0.055* (0.026)	0.063* 0.027	0.066* (0.027)	0.039 (0.028)	0.051 (0.028)	0.038 (0.028)	0.034 (0.029)
Smoking	–0.053*** (0.009)	–0.081*** (0.011)	–0.091*** (0.011)	–0.104*** (0.013)	–0.113*** (0.014)	–0.122*** (0.014)	–0.134*** (0.015)	–0.128*** (0.015)	–0.122*** (0.015)	–0.114*** (0.016)	–0.124*** (0.016)
Colic × Smoking	0.000 (0.023)	0.022 (0.027)	0.019 (0.031)	0.060 (0.032)	0.045 (0.035)	0.083* (0.035)	0.085* (0.036)	0.104** (0.037)	0.075* 0.038	0.050 (0.039)	0.049 (0.040)
Siblings	–0.015* (0.008)	–0.022* (0.009)	–0.012 (0.010)	–0.020 (0.011)	–0.036** (0.012)	–0.046*** (0.012)	–0.044*** (0.012)	–0.040** (0.013)	–0.034* (0.013)	–0.038** (0.014)	–0.035* (0.014)
Constant	0.883*** (0.013)	0.815*** (0.015)	0.783*** (0.016)	0.731*** (0.018)	0.697*** (0.019)	0.656*** (0.020)	0.630*** (0.020)	0.581*** (0.021)	0.563*** 0.022	0.533*** (0.022)	0.508*** (0.022)
*R* ^2^	.041	.062	.062	.066	.066	.070	.073	.066	.060	.057	.059
*N*	4,920	4,920	4,920	4,920	4,920	4,920	4,920	4,920	4,920	4,920	4,920

*Notes:* Robust standard errors are shown in parentheses.

*Source:* DALSC and own calculations on data from Statistics Denmark.

*p < .05; **p < .01; ***p < .001

Couples who had been together longer before having a child had a lower risk of relationship dissolution or divorce, with positive and significant coefficients for log(relationship length) for the entire period. As expected, relationship length also moderates the impact of infantile colic. For the period 1997–2002, the coefficients for the interaction term between log(relationship length) and infantile colic are significant and positive, indicating that infantile colic had a smaller impact on dissolution and divorce risk among couples who had been together longer prior to having a child.

**Fig. 2:**
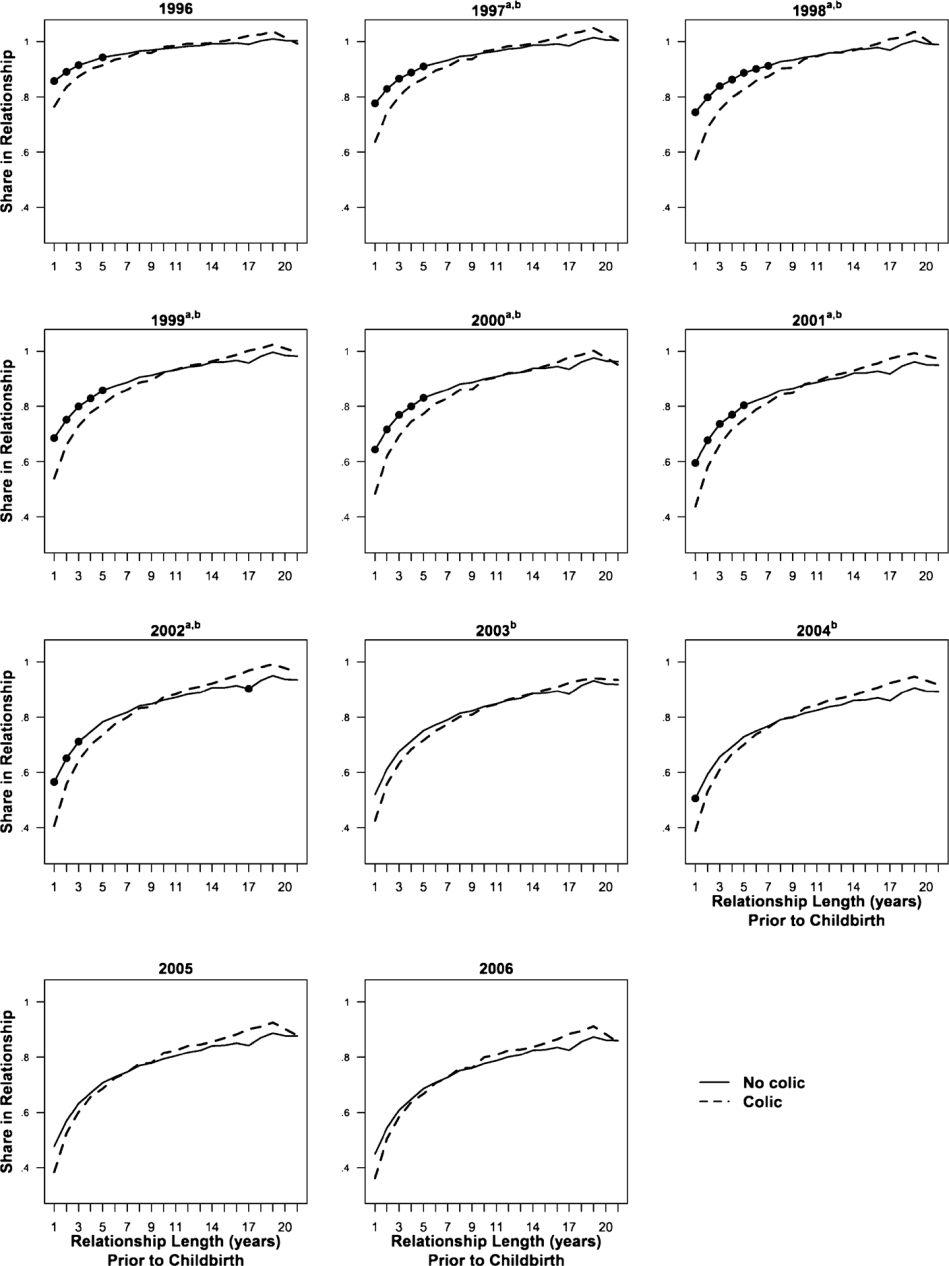
Predicted union rates for couples with versus without an infant with colic across relationship length prior to birth, 1996–2006. *Source:* DALSC and own calculations on data from Statistics Denmark. ^a^Colic indicator significant at 5 % level. ^b^Colic × log(Relationship Length) interaction significant at the 5 % level. Black dots = significant difference (*p* < .05) between point estimates for colic versus no colic

Figure 2 shows the predicted probabilities of being in a relationship for each year and across relationship length prior to childbirth, distinguishing between couples who did and who did not have a colicky child. Infantile colic had the largest impact on couples who had been together for the shortest period but did not have a significant effect on dissolution and divorce risk for couples who had been together for more than seven years. Figure 2 also shows that couples with a colicky child dissolved their relationship at a faster rate from 1996—2002 but that then the couples with noncolicky infants caught up, with only a small and insignificant difference between the groups in rates of dissolution by 2006.

A difference in relationship rates between couples with colicky infants and those with noncolicky babies who were together for only a year before having the child persisted to the end of the study period in 2006. This difference might indicate an overall difference in relationship dissolution rates rather than a shortening of the time until dissolution, but we believe that this is unlikely. The group of couples who had been together for only one year prior to having a child makes up 2 % of the sample (*n* = 107), and the point estimates for them in 2005 and 2006 are not significantly different across colic status. Even if the lack of significance is driven only by the subsample being underpowered, the difference may nevertheless arise through the learning process that we have proposed. Among couples who have been together for only a short time, colic could increase the probability of divorce because it amplifies the effects of the signals that couples receive. It may make compatible couples more susceptible to divorce because of an unlucky sequence of negative signals, whereas the same sequence of signals would not lead to divorce among an otherwise identical compatible couple whose child did not have colic. If this were the case, we should expect to see a difference in union rates between couples who had a colicky infant and those who did not and among those couples who had been together for one year or less. In other words, among couples who had been together for a short time, colic could induce divorce, but this would be as a result of the stochastic element of the information process rather than a permanent change in the relationship.

The validity of our findings could be threatened if couples who were having relationship strain were more likely to report their child as being colicky. However, the fact that Danish infants and their parents receive a number of home visits by a nurse during the child’s infancy—thereby receiving guidance on whether the infant indeed has colic—makes our measure of colic more valid than if the parents had to make the diagnosis on their own. Furthermore, among parents with a colicky infant, the median duration of the colic was 60 days. Only 52 parents with colicky children reported a duration of less than the three-week minimum outlined in the “rule of three.” Treating these cases as not being infantile colic does not change our results. Last, parents with colicky children and those without had identical divorce rates in the five years leading up to the child’s birth, as well as identical relationship rates 10 years after. We therefore think it unlikely that our estimates are biased by any tendency for couples in a strained relationship to be more likely to report their infant as suffering from colic.

### Robustness of the Signal

We conducted a number of additional analyses to test the robustness of infantile colic as an informative signal. We first reran the analysis on a subsample consisting of first-time parents. As noted earlier, the results in Table 2 indicated that first-time parents were more likely to have a colicky child—possibly because either more experienced parents were less likely to report ambiguous cases as colic or because parents whose first child had infantile colic were less likely to have a second child (something we consider later). Table 4 reports the results for the subsample of first-time parents. The estimates for the impact of infantile colic are substantially higher than those shown in Table 3 but statistically significant only for the period 1997–2002, possibly because of the smaller sample size (*N* = 2,067). The coefficients for the interaction term between log(relationship length) and infantile colic are also higher than those reported in Table 3, but again with fewer of them statistically significant (years 1997, 1998, and 2001). Nevertheless, couples who were first-time parents reacted more strongly to infantile colic than did couples who already had children.

**Table 4:** Estimates of union rates for first-time parents with a colicky infant versus those without, 1990–2006

	1996	1997	1998	1999	2000	2001	2002	2003	2004	2005	2006
Colic	–0.081	–0.153*	–0.179*	–0.167*	–0.201**	–0.235**	–0.187*	–0.137	–0.126	–0.115	–0.108
	(0.056)	(0.063)	(0.070)	(0.075)	(0.077)	(0.077)	(0.078)	(0.082)	(0.083)	(0.083)	(0.083)
log(Relationship Length)	0.030**	0.059***	0.070***	0.091***	0.094***	0.108***	0.122***	0.131***	0.128***	0.129***	0.137***
	(0.010)	(0.012)	(0.013)	(0.014)	(0.015)	(0.015)	(0.016)	(0.016)	(0.017)	(0.017)	(0.017)
Colic × log(Relationship Length)	0.037	0.072*	0.074*	0.068	0.070	0.097*	0.078	0.040	0.048	0.053	0.048
	(0.031)	(0.034)	(0.037)	(0.040)	(0.041)	(0.041)	(0.041)	(0.044)	(0.045)	(0.045)	(0.045)
Smoking	–0.070***	0.121***	–0.125***	–0.152***	–0.170***	–0.172***	–0.172***	–0.181***	–0.163***	–0.154***	–0.168***
	(0.015)	(0.019)	(0.020)	(0.022)	(0.023)	(0.024)	(0.024)	(0.025)	(0.026)	(0.026)	(0.026)
Colic × Smoking	–0.001	0.009	0.020	0.068	0.108	0.133*	0.115*	0.171*	0.116	0.069	0.070
	(0.041)	(0.047)	(0.052)	(0.054)	(0.057)	(0.056)	(0.057)	(0.059)	(0.061)	(0.062)	(0.063)
Constant	0.918***	0.855***	0.811***	0.760***	0.741***	0.695***	0.649***	0.606***	0.581***	0.558***	0.527***
	(0.017)	(0.021)	(0.023)	(0.025)	(0.026)	(0.028)	(0.029)	(0.030)	(0.031)	(0.031)	(0.031)
*R* ^2^	.032	.068	.064	.074	.077	.081	.082	.077	.070	.063	.070
*N*	2,067	2,067	2,067	2,067	2,067	2,067	2,067	2,067	2,067	2,067	2,067

*Notes:* Robust standard errors are shown in parentheses.

*Source:* DALSC and own calculations on data from Statistics Denmark.

*p < .05; **p < .01; ***p < .001

#### Testing Alternative Explanations

If infantile colic lowers parents’ subsequent fertility, it would violate our assumption that colic does not cause a persistent change in relationship quality. As Becker et al. (1977) argued, if children function as marriage-specific capital, having additional children may stabilize relationships. However, Danish research has found that children do not stabilize relationships after selection is taken into account (Svarer and Verner 2008). Yet, systematic differences in fertility between couples who had a colicky child and those who did not could still affect the timing of divorce and dissolution. For example, if partners delayed separating until after their youngest child reached a certain age, and if colic affected fertility, differences in fertility patterns could drive the differences in divorce timing rather than differences in the signals’ information content affecting timing.^13^

To test whether colic affected future fertility in our sample, we examine its effect on the number of children that couples had. We do not have direct information on whether the focal child gained new siblings; instead, we use the number of children living in the same household as the focal child who shared at least one biological parent with him or her. We regress this on the right-side variables from Eq. (4), together with the indicator for whether the biological parents were still in a relationship with each other. The latter is entered as both a main effect and interacted with the colic indicator. We also control for whether any children were present at the start of 1995, prior to the birth of the focal child. Table 5 reports the results. Neither the colic indicator, nor any of the interactions with colic, significantly explains the number of children living together with the focal child for any year of the study period. The main effect and the three interactions are also insignificant when tested jointly. Colic does not appear to affect future fertility.

**Table 5:** Estimates of number of children living in same household as focal child for couples with a colicky infant versus those without, with colic interacted with annual parental relationship status, 1997–2006

	1997	1998	1999	2000	2001	2002	2003	2004	2005	2006
Colic	0.015	–0.012	–0.012	0.032	–0.029	–0.010	0.008	0.006	0.028	–0.027
	(0.089)	(0.095)	(0.103)	(0.099)	(0.102)	(0.103)	(0.107)	(0.112)	(0.117)	(0.109)
In Relationship	0.139***	0.226***	0.290***	0.351***	0.330***	0.285***	0.260***	0.263***	0.230***	0.222***
	(0.035)	(0.036)	(0.034)	(0.031)	(0.031)	(0.031)	(0.031)	(0.029)	(0.030)	(0.029)
Colic × In Relationship	0.046	0.072	–0.008	–0.035	0.004	0.063	0.046	0.006	0.000	–0.045
	(0.083)	(0.081)	(0.085)	(0.079)	(0.080)	(0.078)	(0.077)	(0.077)	(0.080)	(0.076)
log(Relationship Length)	0.099***	0.086***	0.048**	0.029	0.005	0.002	–0.016	–0.027	–0.059**	–0.088***
	(0.017)	(0.018)	(0.019)	(0.019)	(0.019)	(0.020)	(0.021)	(0.021)	(0.022)	(0.022)
Colic × log(Relationship Length)	–0.047	–0.052	–0.007	–0.036	–0.023	–0.052	–0.048	–0.028	–0.039	0.004
	(0.043)	(0.046)	(0.048)	(0.050)	(0.051)	(0.052)	(0.053)	(0.052)	(0.055)	(0.054)
Smoking	0.026	–0.023	–0.044	–0.056*	–0.074**	–0.087***	–0.110***	–0.121***	–0.131***	–0.124***
	(0.021)	(0.023)	(0.024)	(0.024)	(0.025)	(0.025)	(0.026)	(0.027)	(0.027)	(0.028)
Colic × Smoking	0.078	0.084	0.030	0.014	0.023	–0.015	–0.040	–0.042	–0.016	–0.023
	(0.058)	(0.061)	(0.062)	(0.061)	(0.060)	(0.062)	(0.063)	(0.065)	(0.069)	(0.069)
Siblings	1.165***	0.925***	0.768***	0.684***	0.617***	0.537***	0.469***	0.403***	0.329***	0.235***
	(0.017)	(0.020)	(0.020)	(0.021)	(0.021)	(0.022)	(0.023)	(0.024)	(0.024)	(0.025)
Constant	0.931***	1.175***	1.399***	1.501***	1.644***	1.768***	1.879***	1.930***	2.043***	2.121***
	(0.039)	(0.040)	(0.040)	(0.039)	(0.040)	(0.041)	(0.042)	(0.042)	(0.042)	(0.043)
*R* ^2^	.508	.364	.274	.234	.192	.146	.111	.088	.058	.037
*N*	4,920	4,920	4,920	4,920	4,920	4,920	4,920	4,920	4,920	4,920

*Notes:* Robust standard errors are shown in parentheses.

*Source:* DALSC and own calculations on data from Statistics Denmark.

*p < .05; **p < .01; ***p < .001

### Quantifying Infantile Colic as an Informative Signal

To examine how informative infantile colic is as a signal, we consider only those couples in our sample who divorced or separated (*N* = 1,734) and regress the length of relationship from childbirth until dissolution on the right-side variables of Eq. (4). This method allows us to gauge how many years of information having a colicky child provides an incompatible couple with (i.e., how informative the signal is), conditioned on relationship length prior to childbirth. Table 6 shows the results.

**Table 6:** Estimation of time in years until divorce among all couples in the sample who divorced, 1996–2006

	Estimated Time Until Divorce (years)
Colic	–1.515**
	(0.559)
log(Relationship Length)	0.626***
	(0.126)
Colic × log(Relationship Length)	0.464
	(0.299)
Smoking	–0.866***
	(0.165)
Colic × Smoking	0.586
	(0.424)
Siblings	0.030
	(0.159)
Constant	4.807***
	(0.224)
*R* ^2^	.051
*F* Value	15.78***
*N*	1,734

*Notes:* Standard deviations are shown in parentheses.

*Source:* DALSC and own calculations on data from Statistics Denmark.

*p < .05; **p < .01; ***p < .001

For couples who had been together one year prior to the focal child’s birth, colic pushed the decision to divorce forward 1.5 years (because log(1) = 0). The effect appears to dwindle as the length of the relationship prior to childbirth increases, although the estimate for the interaction is insignificant. The average couple who dissolved their relationship within the time frame of the data was together for 5.99 years prior to child birth and dissolved their relationship 0.68 years earlier than they would have done had their child not been colicky. Overall, infantile colic has a substantial and significant impact on when couples dissolve their relationship.

## Conclusion

A large literature has studied why people divorce and how lasting shocks to the life situation affect the risk of dissolution. However, much less consideration has been given to the question of how transitory shocks affect divorce risk and how couples reach the decision to divorce. In this article, we developed a Bayesian learning model that describes how transitory shocks to relationships function as informative signals that allow people to assess more quickly whether they are compatible with their partner. Using colic as a natural experiment, we have shown that our model is able to describe the process in which couples with a colicky child divorce faster, albeit not more frequently, than couples without a colicky child. Whereas long-lasting shocks to the life situation—such as changes in income (Weiss and Willis 1997) or having a child with a behavioral disorder (Kvist et al. 2013; Schermerhorn et al. 2012)—appear to incite divorce by changing (information about) living conditions, transitory shocks instead affect the timing of divorce by giving more informative signals about the quality of a relationship. We estimated that colic brought forward divorce by 0.68 years, or approximately 8 months.

We assessed the robustness of our formal model through several tests that allowed the prior level of information about one’s partner to mediate the effect of infantile colic. Taken together, these showed that infantile colic affected the timing of relationship disruption less for couples who already had more information about the quality of their relationship. The findings indicated that our Bayesian learning model, in which couples updated their beliefs’ about their compatibility at each period, could describe how and when couples reached the decision to dissolve their relationship.

We have drawn a sharp distinction between divorce caused by shocks that change the nature of the marital relationship and those that change beliefs about the quality of that relationship. But both can be integrated into the theoretical model that we proposed because shocks to the quality of the relationship itself would lead to change in one or more of *U*(*C*), *U*(*I*) (and thus to the benefit of remaining married, *U*(*M*)), and *U*(*D*)—and, through them, to a shift in the location of the threshold, p*. For example, a shock that increased *U*(*D*) (such as meeting a potential new partner) or reduced *U*(*C*) would cause the threshold to rise, so increasing the likelihood that a run of negative signals would culminate in divorce. Conversely, shocks that increased *U*(*C*) or decreased *U*(*D*) would render the marriage more robust to a run of negative signals. The approach taken in this article, as well as that taken in earlier studies of the causes of divorce, can be considered different special cases of this overarching model.

Our study also has implications for the way we view temporary stressful situations in relationships. The medical literature (e.g., Tanner 2002) has discussed whether medical professionals should pay attention to discord in couples originating from, for example, colicky children. Our results indicate that such transitory shocks may induce short-term strife but need not lead to relationship disruptions that otherwise would not have occurred, given time. Divorcees appear more satisfied after divorce than prior to it (Gardner and Oswald 2006), so there seems little reason to increase support to couples with colicky children if the goal is to keep the relationship intact. Further, a speeding up of divorce may also be beneficial to children in as much as it reduces the time they spend in a stressful domestic environment. More generally, as suggested in our opening epigraph, temporary stressful life changes may have some beneficial effects. By shortening the time to divorce, temporary life changes may more quickly give people the opportunity to find new and compatible partners (see Ananat and Michaels 2008).

## Footnotes

1 Throughout the article, we refer to the process we study as “the timing of divorce.” In our empirical section, however, we study marital divorce and the dissolution of cohabiting unions together. Recent work has demonstrated that cohabiting unions are not only becoming more frequent (Heuveline and Timberlake 2004) but are also becoming more likely to lead to marriage (Reinhold 2010; Svarer 2004) or to resemble marriage (Heuveline and Timberlake 2004; Lundberg 2005; Verbakel and Kalmijn 2014; see, however, Axinn and Thornton 1992). We therefore use the term “divorce” when developing our theoretical model, but we study union dissolution in our empirical application.

2 Previous studies have also referred to this as “match-specific quality” (e.g., Svarer 2004).

3 The official ICD-10 code for the condition is R10.4 (WHO 2010).

4 The presence of nicotine in the mother’s blood through smoking or nicotine patches during pregnancy is the only known predictor (Milidou et al. 2012). We control for smoking in our analysis.

5 We treat the couple as the decision-making unit, and thus we model the beliefs and action of the couple. Although it would be possible to devise a model for the beliefs and actions of each individual within a couple, this would make the model more complex and would not add any further appreciable insight.

6 In reality, these payoffs may not always be fixed. In particular, the payoff from divorce might increase if alternative partners became available, or decrease if the couple gained more marriage specific capital. Changes in *U*(*D*), *U*(*C*), or *U*(*I*) would move the threshold value (discussed in the conclusion) and thereby hasten or delay divorce. We discuss this further in the concluding section.

7 In our model, we fix the rate at which signals arrive, although we could adjust it to allow for signals arriving at faster (slower) rates instead of being more (less) informative. However, because the common sense interpretation of more (less) informative signals is more compelling, we use this form for the model. In terms of timing of divorce, the alternative approach would lead to identical predictions.

8 In this respect, our model aligns with earlier work (e.g., Weiss and Willis 1997), arguing that divorce can occur because of an unpredictable event, but it also develops that approach by addressing the importance of timing. That is, negative unpredictable events can make a compatible couple divorce only when they have received either few signals or a high number of false signals.

9 Certain couples might move in together because of a pregnancy, but such relationships have (at least in the United States) become far less common in recent decades (Stevenson and Wolfers 2007). Because the Nordic countries generally have been on the forefront of progressive changes in living circumstances, this also is likely the case in Denmark.

10 Canivet et al. (2000) compared 4-year-old children who had colic in infancy with a control group who did not. They mainly found no differences in behavioral outcomes between the 50 ex-colicky children and the 102 controls that they observed, but they did find that ex-colicky children are more temperamental. Similarly, Sloman et al. (1990) followed 216 children from birth, finding that colicky children had lower scores on infant development scales at 6 months but that the differences disappeared at later ages.

11 Using the specification test proposed by Mizon and Richard (1986), we find that the log-transformed relationship length fits better than the linear form.

12 Our measure of relationship length is the number of years that couples were together until 1995. Measuring relationship length beyond this point and up to dissolution would introduce endogeneity.

13 We could also view the relationship among childbirth, infantile colic, and later fertility as a set of high-information signals, with the timing of future decisions (e.g., fertility decisions) being a result of high-information signals and those decisions being high-information signals in themselves. However, testing the relationship of such an extensive set of signals is outside the scope of the present study.
